# Novel Brønsted Acid Catalyzed C-C Bond Activation and α-Alkylation of Ketones

**DOI:** 10.3390/molecules29174266

**Published:** 2024-09-09

**Authors:** Wenjuan Li, Huihang Cheng, Huabo Han, Lu Li, Xinming Liu, Xianxu Chu, Xiaopei Li

**Affiliations:** 1 Henan Key Laboratory of Biomolecular Recognition and Sensing, College of Chemistry and Chemical Engineering, Shangqiu Normal University, Shangqiu 476000, China; liwj0523@126.com (W.L.);; 2 Henan Engineering Research Center of Green Synthesis for Pharmaceuticals, School of Chemistry and Chemical Engineering, Shangqiu Normal University, Shangqiu 476000, China

**Keywords:** C-C bond activation, Brønsted acid catalysis, *α*-alkylation of ketone, nucleophilic substitution

## Abstract

A novel approach for the *α*-alkylation of ketones was developed using Brønsted acid-catalyzed C-C bond cleavage. Both aromatic and aliphatic ketones reacted smoothly with 2-substituted 1,3-diphenylpropane-1,3-diones to afford *α*-alkylation products with high yields and with excellent regioselectivity, in which the 1,3-dicarbonyl group acted as a leaving group in the presence of the catalyst TfOH. Mechanism experiments showed that the *β*-C-C bond cleavage of diketone and the shift of the equilibrium towards the enol formation from ketone are driving forces that induce the desired products.

## 1. Introduction

**Scheme 1:**
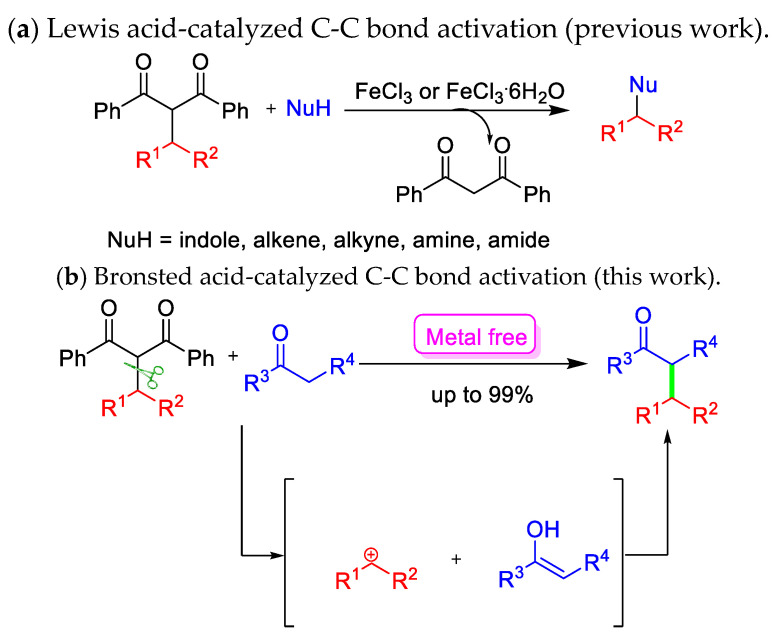
(**a**) Lewis acid-catalyzed C-C bond activation; (**b**) Brønsted acid-catalyzed C-C bond activation.

Selective carbon–carbon bond (C-C bond) activation is a significant strategy in synthetic organic chemistry. It attracts a lot of attention because of its scientific interest and potential utility in organic synthesis [1,2]. C-C bond activation is a new concept that differs from conventional organic synthesis, which involves obtaining the desired molecules by cracking easily obtainable organic materials. It is also a challenge in organic chemistry because C-C bond activation is thermo-dynamically much less favored than C-C bond formation [3,4]. Therefore, many processes have been developed to activate the relatively inert C_sp3_-C_sp3_ bonds. In general, the reported methods are mainly based on using late transition metals, such as rhodium [5,6], ruthenium [7], palladium [8,9], nickel [10], and other metals [11,12,13]. To improve economic performance, some inexpensive and readily abundant metals, such as iron, have also been proven to be efficient in the C-C bond activation reaction [14,15]. In 2008, Li’s group reported FeCl_2_-catalyzed selective C-C bond formation via the oxidative activation of a benzylic C-H bond. Additionally, the group found that C-C bond formation is a reversible process [16]. Then, we reported approaches for C-C bond cleavage to form new C-C bond [17] and C-N bond [18] reactions based on the iron-catalyzed β-C-C bond activation of 1,3-diketones (Scheme 1a). In these works, indole, alkene, alkyne, amine, and amide were used as nucleophiles.

Ketones are weak nucleophiles. To broaden the application of C-C bond activation reactions, ketones, which can be easily converted into enols, were used as nucleophiles. Usually, ketones are transformed into metal enolates [19,20] or enamines [21] and then are reacted with carbon electrophiles to obtain α-alkylated ketones. We propose that the key step in this transformation is the shift in the equilibrium towards enol formation. Thus, adding additives or catalysts to active ketones may promote the desired conversion. Obviously, it is known that the trace residues of transition metals are often difficult to remove from the final products used in pharmaceutical applications [22]. Consequently, it is necessary to develop metal-free selective C-C bond activation reactions. Base or Brønsted acid-catalyzed C-C bond cleavage reactions mainly occur in intramolecular rearrangement reactions [23,24] and tension ring-opening reactions [25]. Therefore, we need to design a proper catalyst system that can activate both C-C bonds and ketone substrates. Based on our previous work and reports from the literature [17,18,26], the β-C-C bond cleavage of ketones can be catalyzed by Lewis acids, and we envision that Brøsted acid may participate in this reaction, which can also promote the conversion from ketone to enol. Hence, we report a novel Brønsted acid-catalyzed C-C bond cleavage and the direct α-alkylation of ketone reactions (Scheme 1b). To the best of our knowledge, the chemistry of the Bronsted acid-catalyzed β-C-C bond activation of carbonyl groups has not been reported. At the same time, this report has a broader substrate scope than previous reports for the α-alkylation of ketones [26].

## 2. Results and Discussion

### 2.1. Optimization Studies

To test our hypothesis, we initiated an investigation on the model reaction of 2- benzhydryl-1,3-diphenylpropane-1,3-dione **1a** and propiophenone **2a** to search for a potential acid catalyst and suitable reaction conditions. The desired product was obtained in a 15% yield in the presence of 20 mol% FeCl_3_ (Table 1, entry 1). The reaction almost stopped when 20 mol% base or amine was added in order to form the enolates or enamines of ketones (Table 1, entries 2–5), probably due to the low activation of the C-C bond. When 20 mol% trifluoromethanesulfonic acid (TfOH) was used as a catalyst with 20 mol% FeCl_3_, the desired product was detected in a 18% yield. Fortunately, when only TfOH was used as a catalyst, **1a** reacted with the 1.0 equivalent of **2a** in DCE at 100 °C for 1 h, and the desired product was obtained in a 52% yield (Table 1, entry 7). MeSO_3_H was less effective for this transformation (Table 1, entry 8), and the desired product was not generated when another weaker Brønsted acid was used, such as TFA, AcOH, and benzoic acid (Table 1, entries 9–11). A reasonable yield of **3a** was detected when BF_4_H was used as a catalyst because of the coordination between the catalyst and **1a** activating the C-C bond (Table 1, entry 12). To our delight, the α-alkylation reaction could be achieved in a higher yield by increasing the amount of **2a** and the catalyst TfOH (Table 1, entries 14 and 15). An excellent result was obtained when the 2 equivalent of **2a** was used with 30 mol% TfOH in DCE at 100 °C for 3 h (Table 1, entry 16). Moreover, other solvents were inferior to DCE with regard to the yield of the desired product, such as THF (<5%), MeCN (60%) and chlorobenzene (49%) (Table 1, entries 17–20). Notably, product **3a** was not observed in the absence of TfOH acid (Table 1, entry 21).

### 2.2. Substrate Scope Studies

With the optimized reaction conditions established, the scope of the present transformation was examined using **2a** as a model substrate to react with various ketones. As shown in Table 2, both aromatic ketones and aliphatic ketones could be effectively transformed into the corresponding product **3**. The aromatic ketones reacted with **1a** efficiently and produced the corresponding compounds **3a** and **3b** in moderate to good yields (Table 2, entries 1–3). The monoalkylation product was highly selectively generated in an excellent yield when **2c** was used in the reaction (Table 2, entry 4). α-Substituted ketone **2d** provided the completely regioselective product **3d** in a good yield. Aliphatic ketones could also be transformed smoothly into the corresponding products in moderate yields by increasing the amount of catalyst with high regioselectivity. Interestingly, the ketone **2g** could also give the α-alkylation product, while the yield of the desired product was not satisfactory.

Subsequently, we examined the scope of ketones with electron-withdrawing groups under the optimized reaction conditions. Various 1,3-dicarbonyl compounds were transformed into the corresponding products **3** with good to excellent yields. To our delight, almost-quantitative yields of the desired products were obtained when 1,3-keto esters were used in the reaction (Table 2, entries 8 and 9). The cyclic 1,3-dicarbonyl substrate could also react with **1a**, giving the corresponding products in moderate yields because of the influence of steric effects (Table 2, entries 10 and 11). Ketones with strong electron-withdrawing groups, such as cyano and sulfonyl, could also afford the corresponding products with excellent yields (Table 2, entries 12 and 13). Furthermore, with both the electron-donating group and electron-withdrawing group on the ring of 2-benzhydryl-1,3-diphenylpropane-1,3-dione **1a**, the corresponding products could be obtained in excellent yields (Table 2, entries 14 and 15).

### 2.3. Proposed Mechanism for Brønsted Acid-Catalyzed C-C Bond Activation

**Scheme 2:**
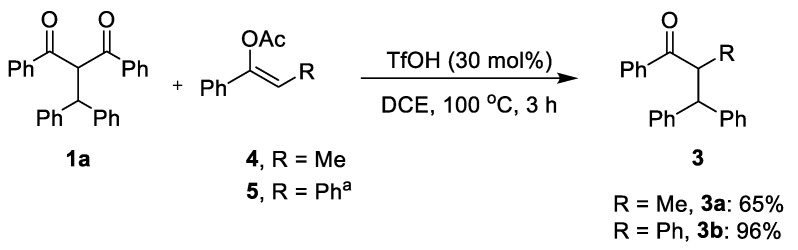
Acid-catalyzed α-alkylation of ketones from enol esters via C-C bond activation. ^a^: 7 h.

To investigate the possible pathways of the present C-C bond cleavage, enol esters, instead of ketones, were reacted with **1a**. Enol esters **4** and **5**, which have stable enol structures, were chosen as substrates [27,28]. α-alkylation product **3a** was obtained in 65% yield and the conversion of **1a** was 66% in the reaction of **1a** with the 2 equivalent of **4**. **3b** was observed in 96% yields in the reaction of **1a** with **5** (Scheme 2). These results indicate that the reaction most likely proceeds through an enol isomer mechanism.

**Scheme 3:**
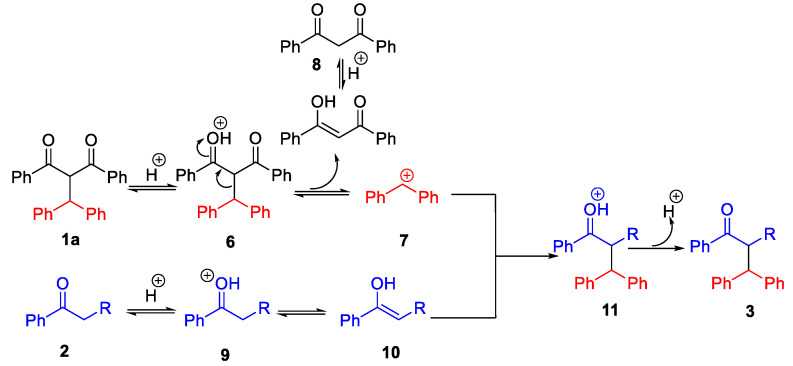
Proposed reaction mechanism.

On the basis of these experimental results, a possible mechanism for the acid-catalyzed C-C bond cleavage and α-alkylation of ketones is shown in Scheme 3. Firstly, 1,3-dicarbonyl compounds **1a** are protonated under strong acid conditions, and enol tautomerization promotes C-C bond cleavage, resulting in the formation of diphenylmethane cations **7**. At the same time, the catalyst TfOH promotes the conversion from ketone to enol **10** through protonation of carbonyl group and the deprotonation of α-H in ketone. Then, **10** can capture **7** to promote the α-alkylation of ketone **11**, subsequently dehydrogenating protons to obtain the target product **3**. Of course, we do not rule out the possibility of electron pairs being present in the dicarbonyl compounds, and this is currently being studied.

## 3. Materials and Methods

### 3.1. General Information

Reagents were purchased from commercial sources and used directly without further purification unless otherwise mentioned. ^1^H NMR (400 MHz) and ^13^C NMR (101 MHz) spectra were recorded on a Bruker 400 spectrometer (Bruker Corporation, Berlin, Germany). Chemical shifts are reported in parts per million (ppm) relative to the internal standard tetramethylsilane (0.00 ppm) or residues of CHCl_3_ (7.26 ppm). The spectra were collected in CDCl_3_. Data are indicated as follows: s = singlet; d = doublet; dd = doublet of doublet; t = triplet; m = multiplet; and q = quartet. Mass spectra (HRMS and ESI-MS) were obtained on an APEX II (Bruker Corporation, Berlin, Germany). IR spectra were collected on a Nicolet 5MX-S infrared spectrometer (Thermo Fisher Scientific, Waltham, MA, USA).

### 3.2. General Experimental Procedure of Reaction of ***1a*** and Enol Ester

To a mixture of 2-benzhydryl-1,3-diphenyl-propane-1,3-dione **1a** (0.2 mmol), (Z)-1-phenylprop-1-en-1-yl acetate **4** (0.4 mmol) in DEC (2 mL), TfOH (0.06 mmol, freshly prepared 0.1 M in DCE) was added under N_2_ at rt. The resulting mixture was stirred at 100 °C for 3 h in a sealed pressure tube. Then, the reaction was cooled to rt. The resulting reaction solution was evaporated in a vacuum to give the crude products. The desired product was purified via flash column chromatography on silica gel using ethyl acetate/petroleum ether (1:50) as an eluent.

### 3.3. General Procedure for Synthesis of Products 3

To a mixture of 2-benzhydryl-1,3-diphenyl-propane-1,3-dione **1a** (0.2 mmol), 1-phenyl-propan-1-one **2a** (0.4 mmol) in DEC (2 mL), TfOH (0.06 mmol, freshly prepared 0.1 M in DCE) was added under N_2_ at room temperature. The resulting mixture was stirred at 100 °C for 3 h in a sealed pressure tube. The desired product was purified via flash column chromatography on silica gel using ethyl acetate/petroleum ether (1:50) as an eluent.

## 4. Conclusions

In summary, we developed a novel and efficient approach for the Brønsted acid-catalyzed β-C-C bond cleavage of carbonyl groups under mild reaction conditions. Both aromatic and aliphatic ketones could participate in the reaction with high yields and excellent regioselectivity. The reversible C-C bond cleavage and equilibrium from ketone to enol are driving forces that induce the desired products. The scope, mechanism, and synthetic application are under investigation.

## Figures and Tables

**Table 1:** Optimization of the reaction conditions ^a^.

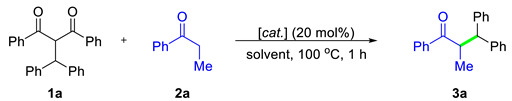				
Entry	2a (equivalent)	Catalyst (20 mol%)	Solvent	Yield (%) ^b^
1	1	FeCl_3_	DCE	15
2	1	FeCl_3_ + Cs_2_CO_3_	DCE	N.D. ^c^
3	1	FeCl_3_ + Na_2_CO_3_	DCE	<5
4	1	FeCl_3_ + NEt_3_	DCE	N.D. ^c^
5	1	FeCl_3_ + L-Proline	DCE	<5
6	1	FeCl_3_ + TfOH	DCE	18
7	1	TfOH	DCE	52
8	1	MeSO_3_H	DCE	<5
9	1	TFA	DCE	N.D. ^c^
10	1	H_3_PO_4_	DCE	N.D. ^c^
11	1	AcOH	DCE	N.D. ^c^
12	1	BF_4_H	DCE	14
13	1	PhCOOH	DCE	N.D. ^c^
14	2	TfOH	DCE	65
15 ^d^	2	TfOH	DCE	76
16 ^e^	2	TfOH	DCE	94
17 ^e^	2	TfOH	THF ^f^	<5
18 ^e^	2	TfOH	DMF	N.D. ^c^
19 ^e^	2	TfOH	MeCN	60
20 ^e^	2	TfOH	PhCl	49
21 ^e^	2	---	DCE	N.D. ^c^

^a^ Reaction conditions: **1a** (0.2 mmol), cat. (0.04 mmol), solvent (3.0 mL), 1 h, N_2_. ^b^ Yields refer to the isolated yield. ^c^ N.D. = not detected using ^1^H NMR. ^d^ TfOH (0.06 mmol). ^e^ TfOH (0.06 mmol), 3 h. ^f^ 80 °C, TfOH (0.06 mmol), 3 h.

**Table 2:** Reactions of 1,3-dicarbonyl compounds and ketones ^a^.

Entry	Substrate	Product	3	Yield (%) ^b^
1	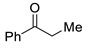	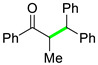	**3a**	94
2	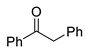	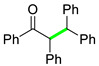	**3b**	61
3	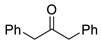	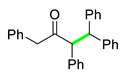	**3c**	97
4	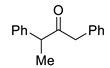	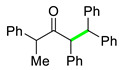	**3d**	55
5	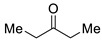	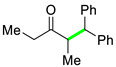	**3e**	48 ^c^
6	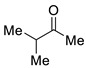	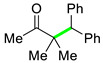	**3f**	42 ^c^
7	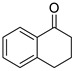	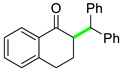	**3g**	43
8	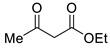	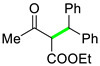	**3h**	97
9	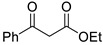	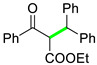	**3i**	90
10	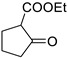	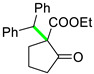	**3j**	57
11	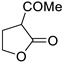	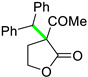	**3k**	57
12	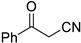	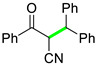	**3l**	96
13	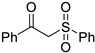	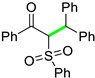	**3m**	85
14	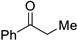	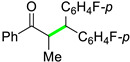	**3n**	87
15	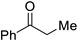	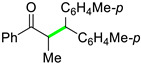	**3o**	90 ^d^

^a^ Conditions: **1** (0.2 mmol), **2** (0.4 mmol), TfOH (0.06 mmol), DCE (3 mL), 100 °C, 3 h. ^b^ Yields refer to the isolated yields. ^c^ TfOH (0.1 mmol). ^d^ 1 h.

## Data Availability

All of the required data are reported in the manuscript and Supplementary Materials.

## Supplementary Materials

The following supporting information can be downloaded at: https://www.mdpi.com/article/10.3390/molecules29174266/s1, Characterization data for obtained products; copies of ^1^H and ^13^C NMR. References [29,30,31,32,33,34,35] are cited in the supplementary materials.
