# Supplementary material for: Novel Brønsted Acid Catalyzed C-C Bond Activation and α-Alkylation of Ketones
Source: Molecules. 2024 Sep 9;29(17):4266. doi: 10.3390/molecules29174266 (PMC11397341; doi:10.3390/molecules29174266)
Supplement: Supplementary file 1 [file molecules-29-04266-s001.zip › molecules-3156082-supplementary.pdf]

Novel Brønsted Acid Catalyzed C-C Bond Activation and α-Alkylation of Ketones

Wenjuan Li [1], Huihang Cheng [1], Huabo Han [1], Lu Li [2], Xinming Liu [2], Xianxu Chu [1] and Xiaopei Li [2,*]

[1] Henan Key Laboratory of Biomolecular Recognition and Sensing, College of Chemistry and Chemical Engineering, Shangqiu Normal University, Shangqiu 476000, China; liwj0523@126.com (W.L.)

[2] Henan Engineering Research Center of Green Synthesis for Pharmaceuticals, School of Chemistry and Chemical Engineering, Shangqiu Normal University, Shangqiu 476000, China

* Correspondence: lixp0813@163.com

Table of contents

1. General Information..........2
2. General procedure for the Brønsted acid catalyzed C-C bond activation..........2
3. The Preparation of 0.1 M Trifluoromethanesulfonic acid (TfOH)..........2
4. Characterizations of compounds **3**..........2
5. NMR spectra for compounds **3**..........7

1. General Information

Reagent were purchased from commercial sources and used as directly without further purification unless mentioned otherwise. ^{1}H NMR (400 MHz) and ^{13}C NMR (101 MHz) spectra are recorded on Bruker 400 MHz spectrometer. Chemical shifts were reported in parts per million (ppm) referenced to tetramethylsilane (0.00 ppm) or residue of $CHCl_3$ (7.26 ppm). The spectra were collected in $CDCl_3$. Data are indicated as follows: s = singlet; d = doublet; dd = doublet of doublet; t= triplet; m = multiplet; q = quartet. Mass spectra (HRMS and ESI-MS) were obtained on APEX II (Bruker Inc.). IR spectra were collected on a Nicolet 5MX-S infrared spectrometer. References [1–28] are cited in the main text.

2. General procedure for Brønsted acid catalyzed C-C bond activation

To a mixture of 2-benzhydryl-1,3-diphenyl-propane-1,3-dione **1a** (0.2 mmol), 1-phenyl-propan-1-one **2a** (0.4 mmol) in DCE (2 mL), TfOH (0.06mmol, freshly prepared 0.1 M in DCE) was added under nitrogen at room temperature. The resulting mixture was stirred at 100 °C for 3 h in a sealed pressure tube. The temperature of reaction was cooled to room temperature. The resulting reaction solution was evaporated in vacuum to give the crude products. Solvent was evaporated and the residue was purified via flash column chromatography on silica gel using ethyl acetate /petroleum ether (1:100) as eluent to afford the pure product **3a** in 94% yield.

3. The Preparation of 0.1 M Trifluoromethanesulfonic acid (TfOH).

TfOH was purchased neat from commercial sources (Alfa Aesar). To a solution of DCE (5 mL), TfOH (0.5 mmol) was added by a syringe at room temperature, string for 5min, then concentration of this mixture was 0.1 M.

4. Characterizations of compounds 3

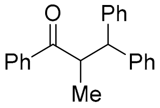

3a

Methyl-1,3,3-triphenylpropan-1-one (3a):[29] Isolated by flash column chromatography (dichloromethane / petroleum ether = 1:1, R_f = 0.6). ^{1}H NMR (400 MHz, $CDCl_3$) δ=7.92(d, J = 8.0 Hz, 2H), 7.55-7.51(m, 1H), 7.45-7.41(m, 2H), 7.36-7.28(m, 4H), 7.25-7.18(m, 3H), 7.11(t, J = 8.0 Hz, 2H), 7.01(t, J = 8.0 Hz, 1H), 4.46-4.37(m, 2H), 1.13(d, J = 5.6 Hz, 3H); ^{13}C NMR (101 MHz, $CDCl_3$) δ = 203.3, 143.5, 143.0, 136.7, 132.9, 128.6, 128.5, 128.4, 128.1, 127.6, 126.5, 126.1, 54.2, 44.8, 18.0.

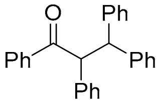

3b

1,2,3,3-Tetraphenylpropan-1-one (3b):[30] Isolated by flash column chromatography (dichloromethane / petroleum ether = 1:1, R_f = 0.6). ^{1}H NMR (400 MHz, $CDCl_3$) δ = 7.93(d, J = 7.2 Hz, 2H), 7.46(d, J = 7.6 Hz, 1H), 7.38-7.32(m, 4H), 7.22-7.18(m, 4H), 7.14-7.00(m, 9H), 5.47(d, J = 12.0 Hz, 1H), 4.49(d, J = 11.6 Hz, 1H); ^{13}C NMR (101 MHz, $CDCl_3$) δ = 198.6, 143.3, 142.4, 137.1, 132.8, 129.0, 128.6, 128.5, 128.4, 128.0, 127.6, 127.0, 126.2, 126.0, 58.2, 55.0.

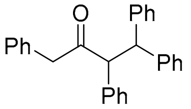 **3c**

1,3,4,4-Tetraphenylbutan-2-one (3c): Isolated by flash column chromatography (dichloromethane / petroleum ether = 1:1, R_f = 0.5). ^{1}H NMR (400 MHz, $CDCl_3$) δ = 7.24-7.22(m, 3H), 7.18-7.11(m, 10H), 7.04-7.86(m, 7H), 4.75(d, J = 12.0 Hz, 1H), 4.71(d, J = 12.0 Hz, 1H), 3.54(s, 2H); ^{13}C NMR (101 MHz, $CDCl_3$) δ = 206.0, 143.0, 142.0, 136.4, 133.5, 130.0, 129.1, 128.6, 128.5, 128.3, 128.1, 127.8, 127.3, 127.0, 126.3, 126.0, 61.7, 53.8, 50.1; IR (neat): ν_{max} 3100, 1668, 1595, 1494, 1449, 1269, 1219, 1028, 983, 771, 694, 570; HRMS (ESI) calcd for $C_{28}H_{24}NaO$ $[M+Na]^+$: 399.1719; found: 399.1723.

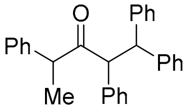 **3d**

1,1,2,4-Tetraphenylpentan-3-one (3d): Isolated by flash column chromatography (dichloromethane / petroleum ether = 1:1, R_f = 0.5).(isomer1 / isomer2 = 1:2) ^{1}H NMR (400 MHz, $CDCl_3$) δ = 7.37-7.26(m, 4H), 7.22-7.12(m, 5H), 7.06-7.00(m, 4H), 6.96-6.95(m, 4H), 6.89-6.87(m, 2H), 6.83-6.81(m, 1H), 4.75-4.60(m, 2H), 3.73(q, J = 6.8 Hz, 0.3H, isomer1), 3.23(q, J = 6.8 Hz, 0.7H, isomer2), 1.15(d, J = 7.2 Hz, 0.9H, isomer1), 1.07(d, J = 7.2 Hz, 2H, isomer2); ^{13}C NMR (101 MHz, $CDCl_3$) δ = 209.8, 207.0, 143.2, 143.0, 142.2, 141.6, 139.7, 139.4, 137.1, 129.4, 129.2, 129.0, 128.8, 128.7, 128.6, 128.4, 128.3, 128.2, 128.1, 127.9, 127.6, 127.2, 127.1, 126.6, 126.5, 126.0, 125.9, 61.8, 61.1, 60.4, 55.0, 53.4, 51.6, 17.5, 17.0; IR (neat): ν_{max} 3026, 2930, 1715, 1599, 1493, 1452, 1288, 1123, 1072, 1030, 746, 725, 698, 530; HRMS (ESI) calcd for $C_{29}H_{27}O$ $[M+H]^+$: 391.2056; found: 391.2062.

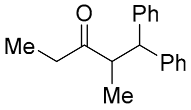 **3e**

2-Methyl-1,1-diphenylpentan-3-one (3e):[31] Isolated by flash column chromatography (dichloromethane / petroleum ether = 1:1, R_f = 0.6). ^{1}H NMR (400 MHz, $CDCl_3$) δ = 7.30-7.28(m, 4H), 7.26-7.22(m, 4H), 7.20-7.16(m, 1H), 7.14-7.09(m, 1H), 4.10(d, J = 11.6 Hz, 1H), 3.49(dq, J = 11.6, 6.8 Hz, 1H), 2.40(dt, J = 21.6, 7.2 Hz, 1H), 2.12(dt, J = 21.6, 7.2 Hz, 1H), 1.01(d, J = 6.8 Hz, 3H), 0.83(t, J = 7.2 Hz, 3H); ^{13}C NMR (101 MHz, $CDCl_3$) δ = 214.6, 143.3, 142.4, 128.6, 128.5, 128.2, 127.7, 126.5, 126.4, 54.7, 50.5, 35.9, 16.7, 7.4 ; IR (neat): ν_{max} 2962, 2926, 1713, 1493, 1450, 1032, 974, 745, 700, 529; HRMS (ESI) calcd for $C_{18}H_{20}NaO$ $[M+Na]^+$: 275.1406; found: 275.1404.

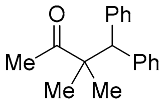 **3f**

3,3-Dimethyl-4,4-diphenylbutan-2-one (3f): Isolated by flash column chromatography (dichloromethane / petroleum ether = 1:1, R_f = 0.6). ^{1}H NMR (400 MHz, $CDCl_3$) δ = 7.26-7.25(m, 8H), 7.22-7.17(m, 2H), 4.41(s, 1H), 2.01(s, 3H), 1.23(s, 6H); ^{13}C NMR (101 MHz, $CDCl_3$) δ = 213.5, 141.5, 129.8, 128.1, 126.5, 58.3, 51.7, 26.0, 23.9; IR (neat): ν_{max} 2957, 2924, 2851, 1703, 1495, 1464, 1449, 1354, 1228, 1109, 769, 702; HRMS (ESI) calcd for $C_{18}H_{20}NaO$ $[M+Na]^+$:

275.1406; found: 275.1407.

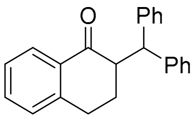

3g

2-Benzhydryl-3,4-dihydronaphthalen-1(2H)-one (3g):[32] Isolated by flash column chromatography (dichloromethane / petroleum ether = 1:1, R_f = 0.5). ^{1}H NMR (400 MHz, $CDCl_3$) δ= 7.92(d, J = 8.0 Hz, 1H), 7.46-7.44(m, 1H), 7.30-7.16(m, 12H), 4.67(d, J = 8.8 Hz, 1H), 3.48(td, J = 9.6,4.0 Hz, 1H), 3.07-2.93(m, 2H), 2.20-2.13(m, 1H), 1.91-1.82(m, 1H); ^{13}C NMR (101 MHz, $CDCl_3$) δ = 199.0, 143.4, 143.2, 142.6, 133.1, 132.9, 128.8, 128.6, 128.5, 128.4, 127.9, 127.6, 126.6, 126.4, 126.3, 51.3, 49.9, 27.8, 26.8; IR (neat): ν_{max} 3059, 3026, 2926, 2870, 1736, 1682, 1599, 1495, 1452, 1288, 1238, 1217, 1155, 1030, 910, 764, 743, 702, 583; HRMS (ESI) calcd for $C_{23}H_{21}O$ [M+H]$^+$: 313.1587; found: 313.1595.

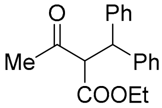

3h

Ethyl 2-benzhydryl-3-oxobutanoate (3h):[33] Isolated by flash column chromatography (ethyl acetate / petroleum ether = 1:10, R_f = 0.6). ^{1}H NMR (400 MHz, $CDCl_3$) δ = 7.30-7.25(m, 8H), 7.18-7.13(m, 2H), 4.77(d, J = 12.4 Hz, 1H), 4.53(d, J = 12.4 Hz, 1H), 4.01-3.94(m, 2H), 2.09(s, 3H), 0.99(t, J = 7.2 Hz, 3H); ^{13}C NMR (101 MHz, $CDCl_3$) δ = 201.7, 167.6, 141.5, 141.2, 128.8, 128.5, 127.7, 127.6, 126.9, 126.8, 65.1, 61.4, 50.8, 30.0, 13.7, 1.0.

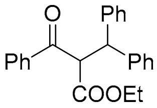

3i

Ethyl 2-benzhydryl-3-oxo-3-phenylpropanoate (3i):[17] Isolated by flash column chromatography (ethyl acetate / petroleum ether = 1:10, R_f = 0.6). ^{1}H NMR (400 MHz, $CDCl_3$) δ = 8.03-8.01(m, 2H), 7.54(t, J = 7.2 Hz, 1H), 7.44-7.37(m, 4H), 7.30-7.23(m, 4H), 7.20-7.12(m, 3H), 7.05(t, J = 8.4 Hz, 1H), 5.42(d, J = 12.0 Hz, 1H), 5.09(d, J = 12.0 Hz, 1H), 3.97-3.85(m, 2H), 0.92(t, J = 7.2 Hz, 3H); ^{13}C NMR (101 MHz, $CDCl_3$) δ = 129.8, 167.7, 141.6, 136.5, 133.6, 128.6, 128.5, 128.2 127.6, 126.8, 126.5, 61.5, 59.4, 50.8, 13.7.

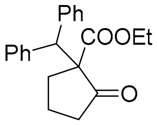

3j

Methyl 1-benzhydryl-2-oxocyclopentanecarboxylate (3j):[34] Isolated by flash column chromatography (ethyl acetate / petroleum ether = 1:10, R_f = 0.6) ^{1}H NMR (400 MHz, $CDCl_3$) δ = 7.30-7.24(m, 4H), 7.22-7.14(m, 4H), 7.09-7.07(m, 2H), 5.26(s, 2H), 3.33(s, 3H), 3.08-3.03(m, 1H), 2.31-2.23(m, 2H), 1.95-1.80(m, 1H), 1.76-1.67(m, 1H), 1.53-1.45(m, 1H); ^{13}C NMR (101 MHz, $CDCl_3$) δ = 214.0, 169.3, 141.1, 140.2, 130.1, 128.7, 128.4, 128.3, 126.8, 126.5, 66.2, 55.0, 52.7, 38.6, 29.3, 19.7; IR (neat): ν_{max} 3028, 2953, 1749, 1724, 1495, 1450, 1259, 1229, 1138, 1105, 939, 704, 534.

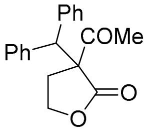

3k

2-**Acetyl-3-benzhydryldihydrofuran-2(3H)-one (3k):** Isolated by flash column chromatography (ethyl acetate / petroleum ether = 1:10, R_f = 0.6). ^{1}H NMR (400 MHz, $CDCl_3$) δ = 7.34-7.30(m, 2H), 7.28-7.22(m, 4H), 7.17-7.13(m, 4H), 5.38(s, 1H), 4.08(q, J = 8.0 Hz, 1H), 3.62(td, J = 8.8, 4.0 Hz, 1H), 3.38-3.31(m, 1H), 2.42-2.35(m, 1H), 2.21(s, 3H); ^{13}C NMR (101 MHz, $CDCl_3$) δ = 201.2, 174.9, 139.3, 139.0, 129.6, 129.0, 128.8, 128.7, 127.4, 127.3, 67.0, 66.5, 54.1, 25.8, 25.5; IR (neat): ν_{max} 2924, 2359, 2330, 1757, 1705, 1495, 1447, 1157, 1028, 953, 731, 700; HRMS (ESI) calcd for $C_{19}H_{22}NO_3[M+NH_4]^+$: 312.1594; found: 312.1600.

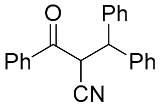

3l

2-Benzhydryl-3-oxo-3-phenylpropanenitrile (3l*)*:[35] Isolated by flash column chromatography (ethyl acetate / petroleum ether = 1:10, R_f = 0.6). ^{1}H NMR (400 MHz, $CDCl_3$) δ = 7.89-7.87(m, 2H), 7.60(t, J = 7.2 Hz, 1H), 7.46(t, J = 8.6 Hz, 2H), 7.37-7.32(m, 4H), 7.29-7.25(m, 1H), 7.24-7.19(m, 4H), 7.16-7.12(m, 1H), 5.18(d, J = 9.6 Hz, 1H), 4.89(d, J = 9.6 Hz, 1H); ^{13}C NMR (101 MHz, $CDCl_3$) δ = 189.8, 139.7, 139.4, 134.7, 134.4, 129.0, 128.9, 128.8, 128.6, 128.3, 127.7, 127.6, 127.4, 116.3, 50.8, 44.4; IR (neat): ν_{max} 3063, 3030, 2243, 1694, 1595, 1495, 1449, 1323, 1265, 1231, 1084, 1001, 748, 698, 623; HRMS (ESI) calcd for $C_{22}H_{17}NO$ $[M+H]^+$: 312.1383; found: 312.1392.

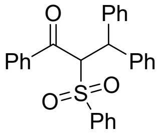

3m

1,3,3-Triphenyl-2-(phenylsulfonyl)propan-1-one (3m): Isolated by flash column chromatography (ethyl acetate / petroleum ether = 1:2, R_f = 0.5). ^{1}H NMR (400 MHz, $CDCl_3$) δ = 7.83-7.81(m, 2H), 7.55-7.47(m, 4H), 7.43-7.39(m, 2H), 7.35-7.29(m, 4H), 7.18-7.12(m, 5H), 7.04-6.94(m, 3H), 6.14(d, J = 12.0 Hz, 1H), 4.86(d, J = 12.0 Hz, 1H); ^{13}C NMR (101 MHz, $CDCl_3$) δ = 192.2, 140.6, 139.2, 137.9, 133.6, 133.5, 129.2, 128.7, 128.6, 128.5, 128.4, 128.3, 128.2, 77.2, 52.0; IR (neat): ν_{max} 3426, 3387, 2253, 2126, 1672, 1659, 1450, 1302, 1273, 1142, 1051, 1026, 1007, 826, 764, 629, 554; HRMS (ESI) calcd for $C_{27}H_{22}NaO_3S$ (M+Na): 449.1182; found: 449.1194.

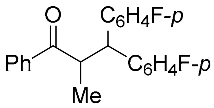

3n

3,3-Bis(4-fluorophenyl)-2-methyl-1-phenylpropan-1-one (3n): Isolated by flash column chromatography (ethyl acetate / petroleum ether = 1:10, R_f = 0.5). ^{1}H NMR (400 MHz, $CDCl_3$) δ = 7.91-7.89 (m, 2H), 7.56(t, J = 8.0 Hz, 1H), 7.45(t, J = 8.0 Hz, 2H), 7.29-7.27(m, 2H), 7.18-7.16(m, 2H), 7.01 (t, J = 8.0 Hz, 2H), 6.81(t, J = 8.0 Hz, 2H); ^{13}C NMR (101 MHz, $CDCl_3$) δ = 203.0, 162.8, 162.4, 160.3, 160.0, 129.2, 129.1, 136.6, 133.2, 129.8, 129.7, 129.0, 128.9, 128.7,

128.0, 52.7, 45.1, 17.9.

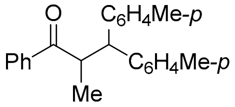

3o

2-**Methyl-1-phenyl-3,3-di-p-tolylpropan-1-one (3o):** Isolated by flash column chromatography (ethyl acetate / petroleum ether = 1:10, R_f = 0.5). ^{1}H NMR (400 MHz, $CDCl_3$) δ = 7.94-7.92(m, 2H), 7.58-7.52(m, 1H), 7.45-7.42(m, 2H), 7.22 (d, *J* = 8.0 Hz, 2H), 7.15-7.06(m, 4H), 6.91(d, *J* = 8.0 Hz, 2H), 4.41-4.32(m, 2H), 2.30(s, 3H), 2.16(s, 3H), 1.12(d, *J* = 8.0 Hz, 3H). ^{13}C NMR (101 MHz, $CDCl_3$) δ = 203.5, 140.9, 140.3, 136.8, 135.9, 135.4, 132.9, 129.3, 129.1, 128.6, 128.2, 128.1, 127.4, 53.4, 44.8, 20.9, 20.8, 18.0.

References:

1. Park, Y.J.; Park, J.-W.; Jun, C.-H. Metal-Organic Cooperative Catalysis in C-H and C-C Bond Activation and Its Concurrent Recovery. *Acc. Chem. Res*. **2008**, *41*, 222 - 234.
2. Jun, C.-H. Transition metal-catalyzed carbon-carbon bond activation. *Chem. Soc. Rev.* **2004**, *33*, 610 - 618.
3. Crabtree, R.H. The organometallic chemistry of alkanes. *Chem. Rev*. **1985**, *85*, 245 - 269.
4. Rybtchinski, B.; Milstein, D. Metal Insertion into C-C Bonds in Solution. *Angew. Chem. Int. Ed.* **1999**, *38*, 870 - 883.
5. Paul, T.; Basak, S.; Punniyamurthy, T. Weak Chelation-Assisted C4-Selective Alkylation of Indoles with Cyclopropanols via Sequential C-H/C-C Bond Activation. *Org. Lett*. **2022**, *24*, 6000 - 6005.
6. Calow, A.D.J.; Dariller, D.; Bower, J.F. Carbonylative N-Heterocyclization via Nitrogen-Directed C-C Bond Activation of Nonactivated Cyclopropanes. *J. Am. Chem. S*oc. **2022**, *144*, 11069 - 11074.
7. Kondo, T.; Kaneko, Y.; Taguchi, Y.; Nakamura, A.; Okada, T.; Shiotsuki, M.; Ura, Y.; Wada, K.; Mitsudo, T. Rapid Ruthenium-Catalyzed Synthesis of Pyranopyrandiones by Reconstructive Carbonylation of Cyclopropenones Involving C-C Bond Cleavage. *J. Am. Chem. Soc.* **2002**, *124*, 6824 - 6825.
8. Chen. L.; Shi, C.; Li, W.; Li, B.; Zhu, J.; Lin, A.; Yao, H. Palladium-Catalyzed Asymmetric C-C Bond Activation/Carbonylation of Cyclobutanones. *Org. Lett*. **2022**, *24*, 9157 - 9162.
9. Chiba, S.; Xu, Y.-J.; Wang, Y.-F. A Pd(II)-Catalyzed Ring-Expansion Reaction of Cyclic 2-Azidoalcohol Derivatives: Synthesis of Azaheterocycles. *J. Am. Chem. Soc*. **2009**, *131*, 12886 - 12887.
10. Ding, D.; Lan, Y.; Lin, Z.; Wang, C. Synthesis of gem-Difluoroalkenes by Merging Ni-Catalyzed C-F and C-C Bond Activation in Cross-Electrophile Coupling. *Org. Lett.* **2019**, *21*, 2723 - 2730.
11. Choi, S.-M.; Park, J.-U.; Kim, J.H. CoIII-Catalyzed C-H Alkenylation and Allylation with Cyclopropenes via Sequential C-H/C-C Bond Activation. *Org. Lett*. **2021**, *23*, 6674 - 6679.
12. Shi, S.-H.; Liang, Y.; Jiao, N. Electrochemical Oxidation Induced Selective C-C Bond Cleavage. *Chem. Rev*. **2021**, *121*, 485 - 505.
13. Yu, X.-Y.; Chen, J.-R.; Xiao, W.-J. Visible Light-Driven Radical-Mediated C-C Bond Cleavage/Functionalization in Organic Synthesis. *Chem. Rev*. **2021**, *121*, 506 - 561.

14. Yu, Q.; Ma, S. Lewis acid-catalyzed unexpected selective C-C bond cleavage: An efficient and mild construction of cyclopentenes. *Chem. Commun.* **2012**, *48*, 11784–11786.
15. Dieskau, A.P.; Holzwarth, M.S.; Plietker, B. Fe-Catalyzed Allylic C–C-Bond Activation: Vinylcyclopropanes As Versatile a1,a3,d5-Synthons in Traceless Allylic Substitutions and [3 + 2]-Cycloadditions. *J. Am. Chem. Soc.* **2012**, *134*, 5048–5051.
16. Li, Z.; Cao, L.; Li, C.-J. FeCl2-Catalyzed Selective C-C Bond Formation by Oxidative Activation of a Benzylic C-H Bond. *Angew. Chem. Int. Ed.* **2007**, *46*, 6505–6507.
17. Li, H.; Li, W.; Liu, W.; He, Z.; Li, Z. An Efficient and General Iron-Catalyzed C-C Bond Activation with 1,3-Dicarbonyl Units as a Leaving Groups. *Angew. Chem. Int. Ed.* **2011**, *50*, 2975–2978.
18. Li, W.; Zheng, X.; Li, Z. Iron-Catalyzed C-C Bond Cleavage and C-N Bond Formation. *Adv. Synth. Catal.* **2013**, *355*, 181–190.
19. Casiraghi, G.; Battistini, L.; Curti, C.; Rassu, G.; Zanardi, F. The Vinylogous Aldol and Related Addition Reactions: Ten Years of Progress. *Chem. Rev.* **2011**, *111*, 3076–3154.
20. Palomo, C.; Oiarbide, M.; Garcıa, J.M. Current progress in the asymmetric aldol addition reaction. *Chem. Soc. Rev.* **2004**, *33*, 65–75.
21. Mlynarski, J.; Gut, B. Organocatalytic synthesis of carbohydrates. *Chem. Soc. Rev.* **2012**, *41*, 587–596.
22. Magano, J.; Dunetz, J.R. Large-Scale Applications of Transition Metal-Catalyzed Couplings for the Synthesis of Pharmaceuticals. *Chem. Rev.* **2011**, *111*, 2177–2250.
23. Popp, F.D.; McEwen, W.E. Polyphosphoric Acids As A Reagent In Organic Chemistry. *Chem. Rev.* **1958**, *58*, 321–401.
24. Linsk, J. Rearrangement of 4-Methylcyclohexene during Sulfuric Acid-catalyzed Reaction with Benzene. *J. Am. Chem. Chem. Soc.* **1950**, *72*, 4257–4260.
25. De Los Ríos, C.; Hegedus, L.S. Reaction of Optically Active α-Aminoallenylstannanes with Aldehydes Formed in Situ from the Lewis-Acid-Catalyzed Rearrangement of Epoxides. *J. Org. Chem.* **2005**, *70*, 6541–6543.
26. Koppolu, S.R.; Naveen, N.; Balamurugan, R. Triflic Acid Promoted Direct α-Alkylation of Unactivated Ketones Using Benzylic Alcohols via in Situ Formed Acetals. *J. Org. Chem.* **2014**, *79*, 6069–6078.
27. Hokamp, T.; Wirth, T. Hypervalent Iodine(III)-Catalysed Enantioselective α-Acetoxylation of Ketones. *Chem. Eur. J.* **2020**, *26*, 10417–10421.
28. Basdevant, B.; Legault, C.Y. Enantioselective Iodine(III)-Mediated Synthesis of α-Tosyloxy Ketones: Breaking the Selectivity Barrier. *Org. Lett.* **2015**, *17*, 4918–4921.
29. Jayamani, M.; Pant, N.; Ananthan, S.; Narayanan, K.; Pillai, C.N. Synthesis of indenes from phenylpropanones using alumina catalyst. *Tetrahedron* **1986**, *42*, 4325–4332.
30. Zimmerman, H.E.; Nuss, J.M.; Tantillo, A.W. Cyclopropanols and the Di-ϖ-methane Rearrangement: Mechanistic and Exploratory Organic Photochemistr. *J. Org. Chem.* **1988**, *53*, 3792–3803.
31. Tandiary, M.A.; Asano, M.; Hattori, T.; Takehira, S.; Masui, Y.; Onaka, M. Unprecedented alkylation of silicon enolates with alcohols via carbenium ion formations catalyzed by tin hydroxide-embedded montmorillonite. *Tetrahedron Lett.* **2017**, *58*, 1925-1928.
32. Yang, C.-F.; Wang, J.-Y.; Tian, S.-K. Catalytic decarboxylative alkylation of β-keto acids with sulfonamides via the cleavage of carbon–nitrogen and carbon–carbon bonds. *Chem.*

Commun. **2011**, *47*, 8343 – 8345.
33. Yasuda, M.; Somyo, T.; Baba, A. Direct carbon-carbon bond formation from alcohols and active methylenes, alkoxyketones, or indoles catalyzed by indium trichloride. *Angew. Chem. Int. Ed*. **2006**, *45*, 793 – 796.
34. Babu, S.A.; Yasuda. M.; Tsukahara, Y.; Yamauchi, T.; Wada, Y.; Baba, A. Microwave-irradiated transition-metal catalysis: rapid and efficient dehydrative carbon-carbon coupling of alcohols with active methylenes. *Synthesis* **2008**, *11*, 1717 – 1724.
35. Sondengam, B.L.; Fomum, Z.T.; Charles, G.; Akam, T.M. A Convenient Reduction of Activated Olefens by Zinc-Copper Couple. *J. Chem. Soc. Perkin trans I.* **1983**, *6*, 1219 – 1222.

5. **NMR spectra for compounds 3.**

^{1}H NMR of **3f**

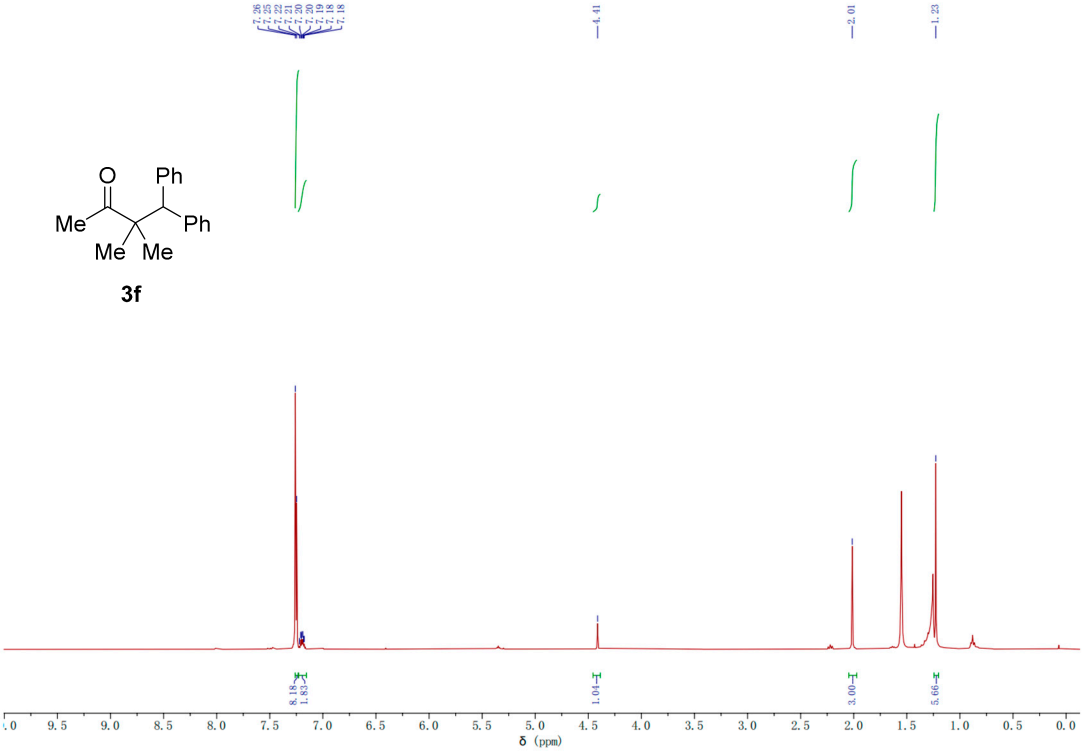

^{13}C NMR of **3f**

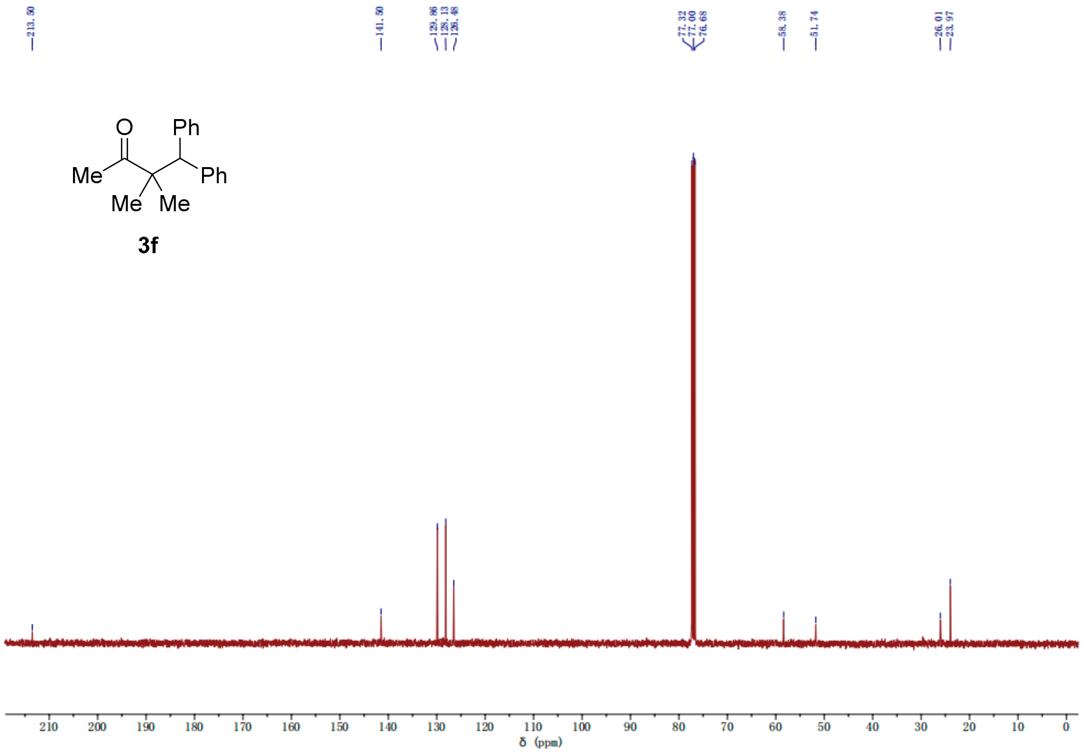

^{1}H NMR of **3k**

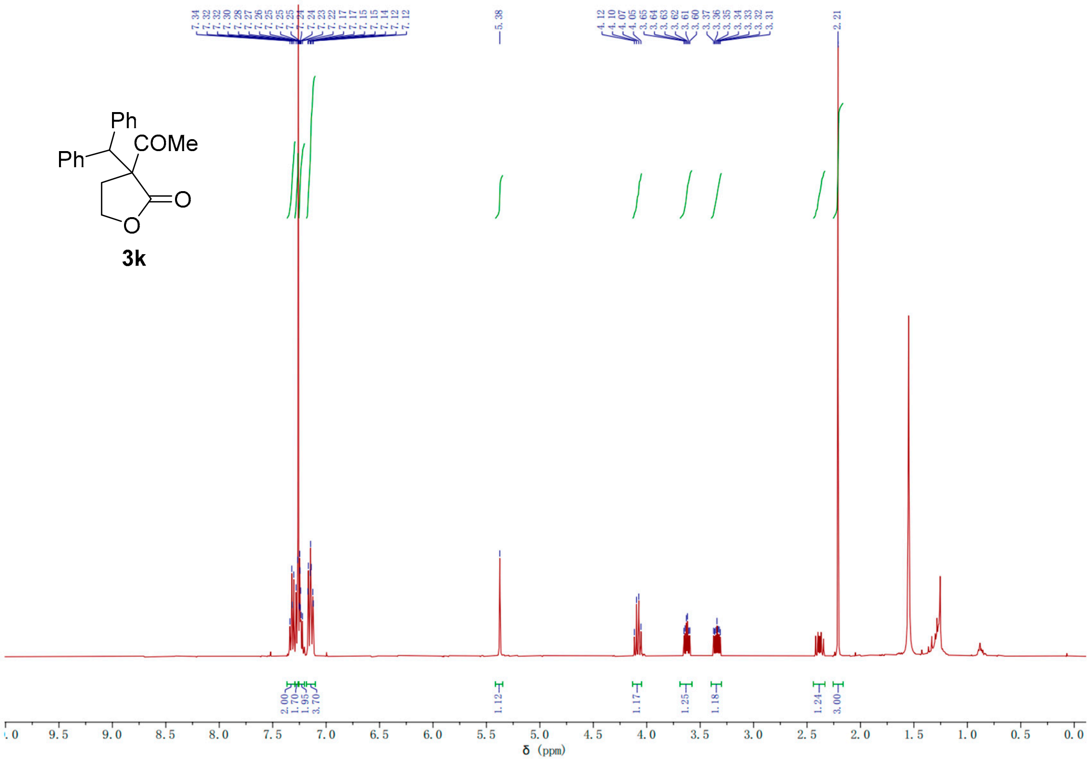

^{13}C NMR of **3k**

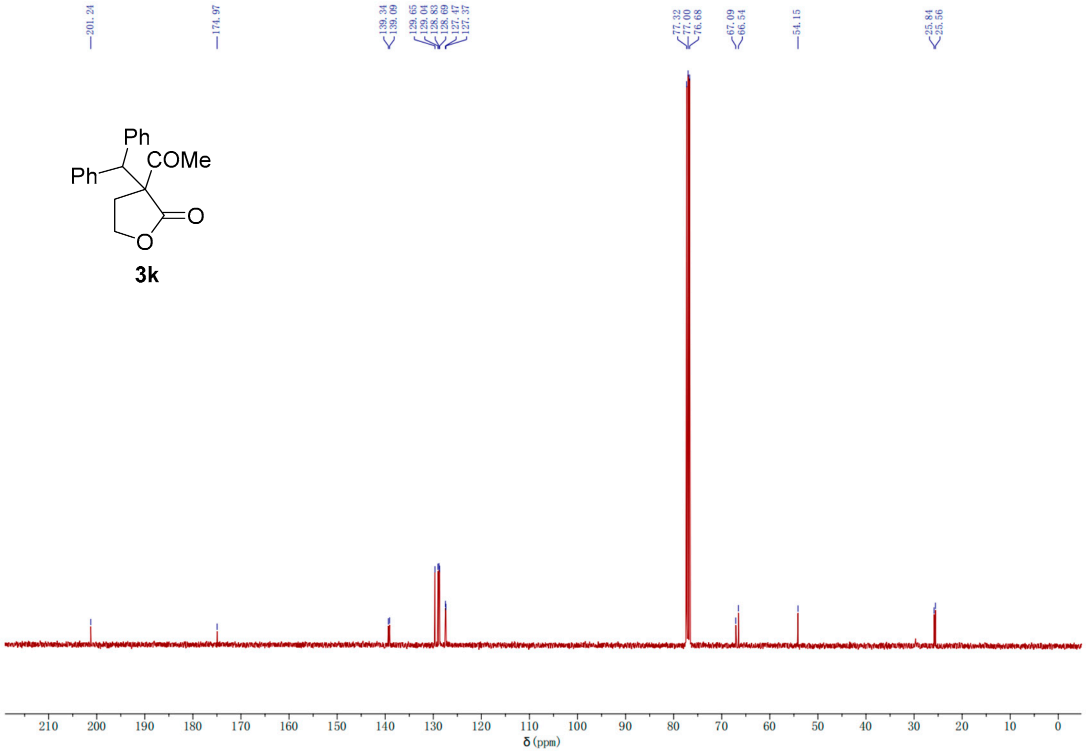

^{1}H NMR of **3m**

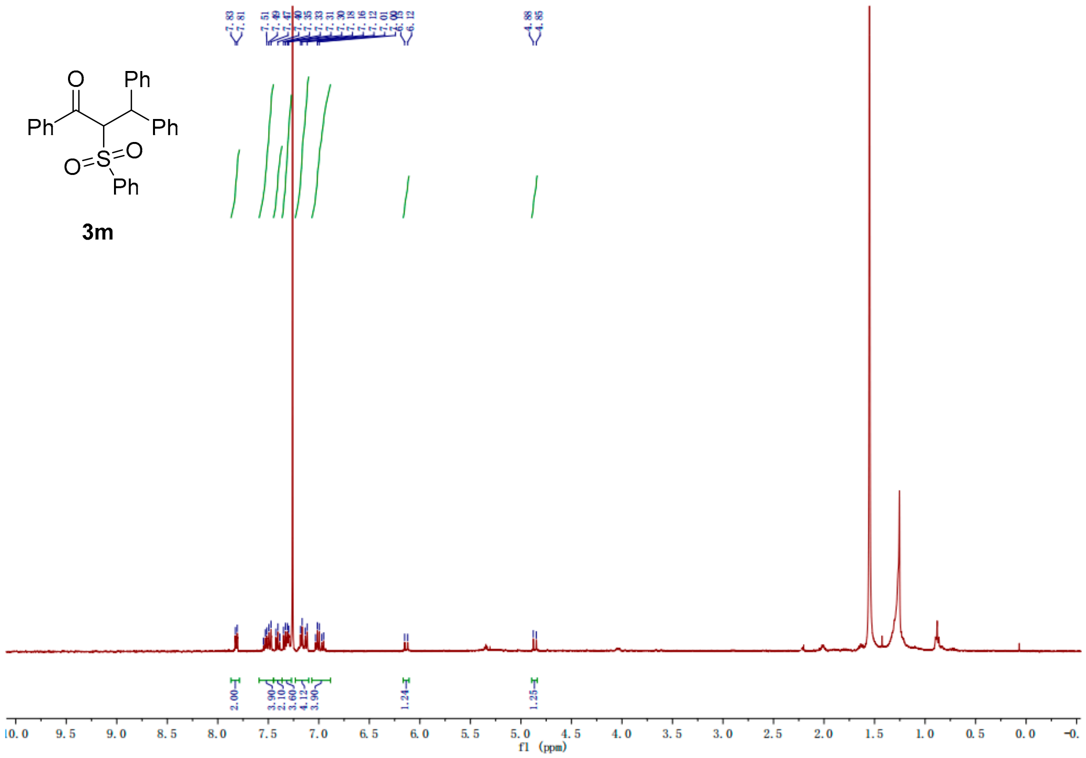

^{13}C NMR of **3m**

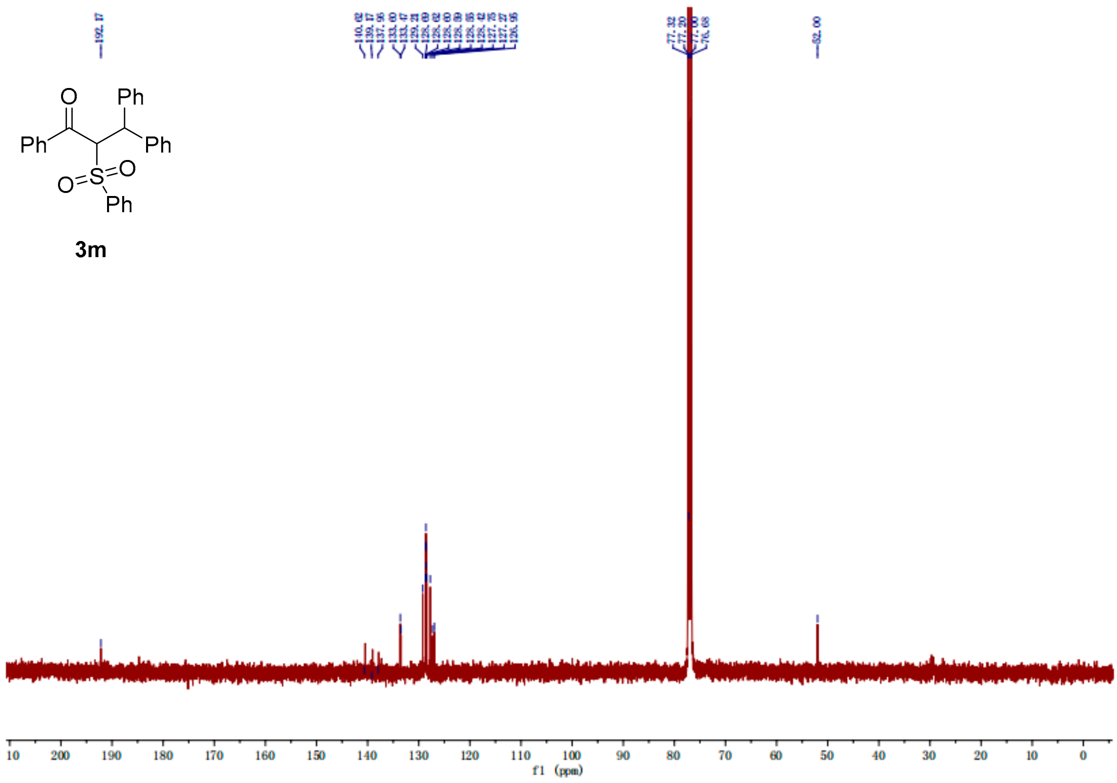

Enlarging chemical shift of ^{13}C NMR of **3m** from 76.68 to 77.20.

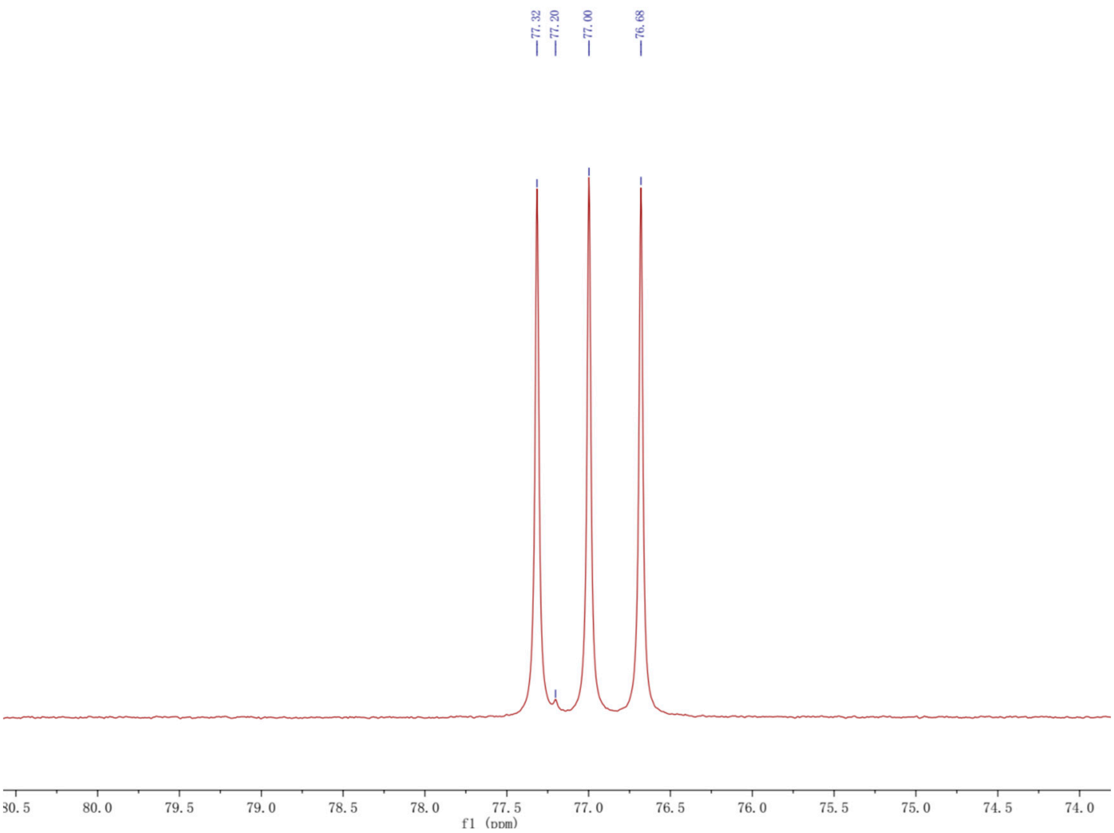

^1H NMR of **3n**

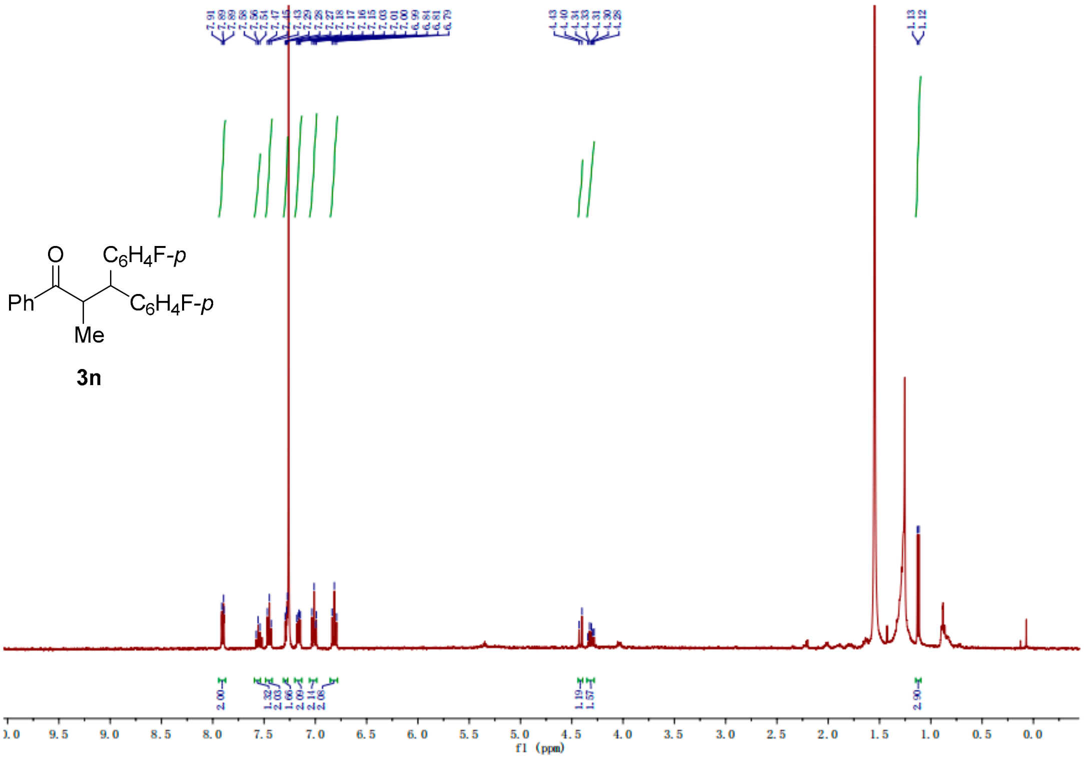

^{13}C NMR of **3n**

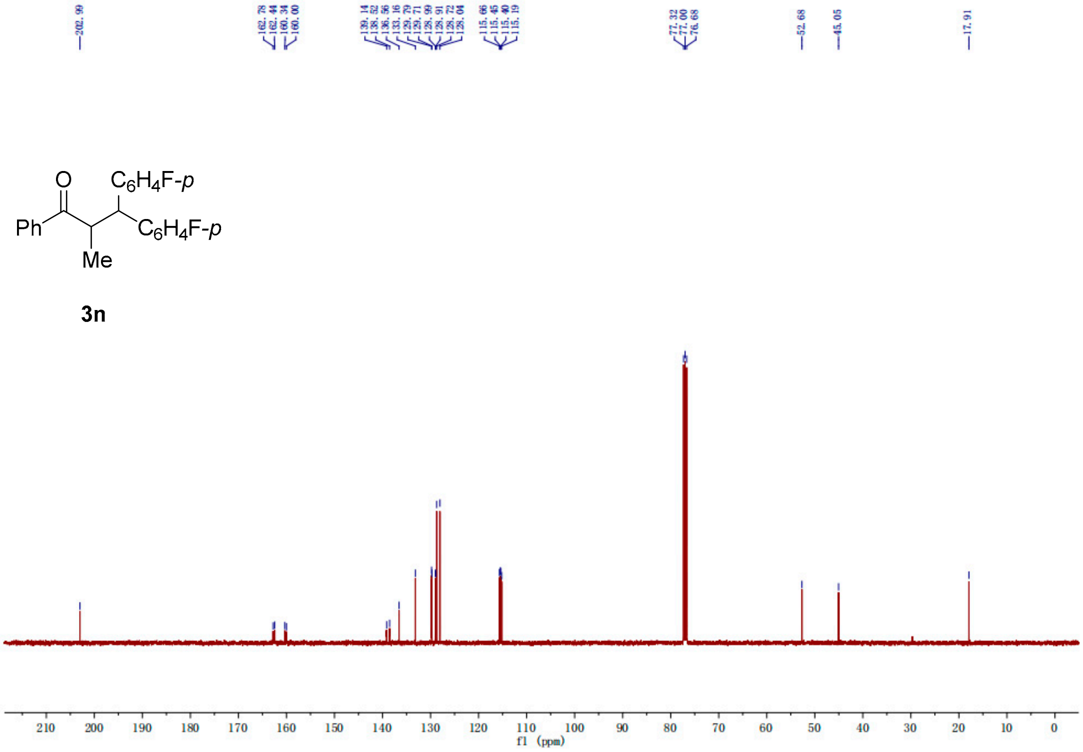

^{1}H NMR of **3o**

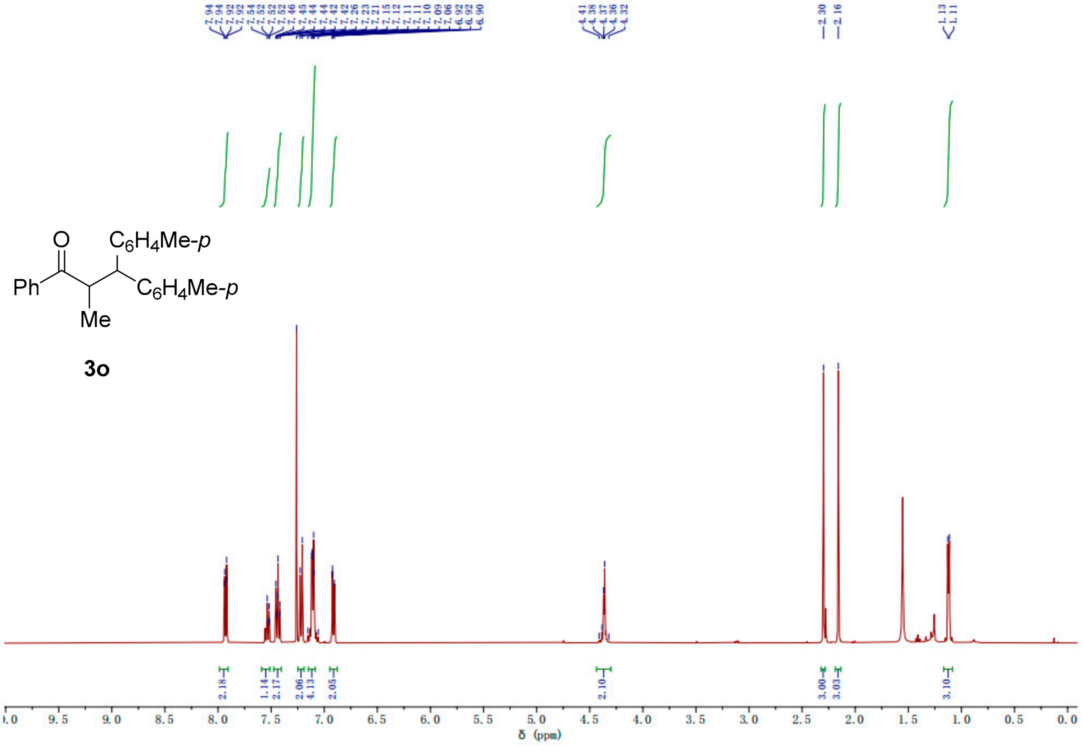

^{13}C NMR of **3o**

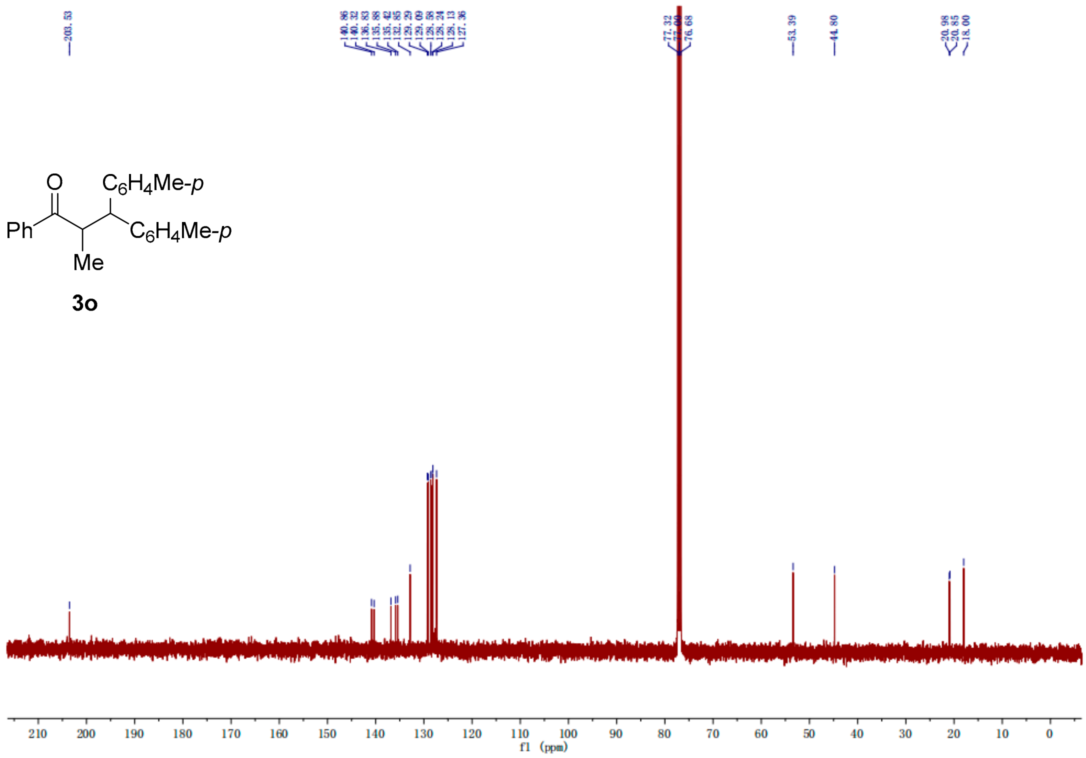

203.53
140.86
140.32
136.83
135.88
135.42
132.85
129.29
129.09
128.58
128.24
128.13
127.36
77.32
77.00
76.68
53.39
44.80
20.98
20.85
18.00
O
C6H4Me-p
Ph
C6H4Me-p
Me
3o
210 200 190 180 170 160 150 140 130 120 110 100 90 80 70 60 50 40 30 20 10 0
f1 (ppm)
